# Why are keratins important?

**DOI:** 10.1186/s40246-022-00379-y

**Published:** 2022-01-30

**Authors:** Jeffrey Nicholas Fisk, Daniel W. Nebert

**Affiliations:** 1 Program of Computational Biology and Bioinformatics, Yale University, New Haven, CT 06511 USA; 2 Departments of Pediatrics and Molecular and Developmental Biology, Cincinnati Children’s Research Center, Cincinnati, OH 45229 USA; 3 Department of Environmental Health and Center for Environmental Genetics, University of Cincinnati College of Medicine, Cincinnati, OH 45267 USA

The human fertilized egg (*zygote*) is probably the most implausible cell on Earth, because it is equipped with all the genes needed to create a human body—comprising ~ 37 trillion cells and representing more than 200 distinctly different cell-types. This complexity emerges from how and when the regulatory genes become activated to express the essential structural genes at the right time during embryogenesis, fetogenesis, postpartum, and all the way to adulthood.

How does that 1-cell zygote hold itself together? How do the nucleus and nucleolus remain intact from the cytoplasm, and how do all the cytoplasmic organelles persist as distinct subcellular bodies? Likewise, how do all the zygote’s successor cells hold themselves together, despite their diversification into > 200 cell-types that are localized uniquely into dozens of specific tissues? The answer, in part, lies in (regulatory) genes involved in signaling and adhesion, and formation of filaments and fibrils and their (structural) gene products (proteins). If these types of *Animalia* proteins had not evolved (with orgins as early as the the first eukaryote) to hold cells together and keep cells organized within a particular tissue, life on Earth would be drastically different.

One large subset of these genes responsible for keeping everything in their place is the Intermediate Filament (IntFil) gene superfamily. When we were first invited to join as coauthors on the Ho et al*.* [1] project, we knew nothing about IntFil genes and their proteins, nor why anyone would want to study them.

IntFils arose during early metazoan evolution to provide mechanical support for plasma membranes that are connected and interact with other cells and the extracellular matrix. IntFils are ubiquitous structural components that comprise, in a cell type-specific manner, the cytoskeleton infrastructure in all animal tissues. All IntFil proteins show a distinctly organized extended α-helical conformation, which is predisposed to form two-stranded coiled coils that reflect the basic building blocks of highly flexible, stress-resistant cytoskeletal filaments. In this issue, Ho et al*.* [1] studied the evolutionary history of IntFil genes. Although IntFils are divided into six types, the coauthors focused on the type I “acidic” and type II “basic” keratin genes—which are much larger in number and evolutionarily emerged more recently than the other four types.

The first keratin gene appeared in sponge, three keratin genes are found in arthropods, and then more rapid increases in keratin genes occurred in lungfish and amphibian genomes, concomitant with the sea animal-to land animal transition which occurred 440 to 410 million years ago. The human genome has 27 of 28 type I keratin genes clustered at chromosome (Chr) 17q21.2, and all 26 type II keratin genes clustered at Chr 12q13.13. The mouse genome has 27 of 28 type I keratin genes clustered on Chr 11, and all 26 type II clustered on Chr 15; all the mouse keratin genes are syntenic with the human keratin genes. On the other hand, the zebrafish genome has 18 type I keratin genes scattered on five chromosomes and three type II keratin genes on two chromosomes. The two clusters (“evolutionary blooms”) of type I and type II keratin genes, each located along a chromosomal segment, have been found in all seven nonhuman mammalian genomes that have been examined to date, but not in fish genomes [1].

Screening 259 species and subspecies in 20 phyla of animals, from jellyfish to human, Ho et al*.* [1] examined various features found in the type I and type II keratin proteins. They found evidence that some genes appear to have arisen in an early species, disappeared in a later species, and then, on occasion, reappeared and apparently were repurposed to provide for new features in more recently diverged species.

To create the maximum-likelihood trees, Ho et al*.* [1] aligned sequences in *MAFFT* [2] using the *L-INS-i* local pair algorithm [3] with 10,000 iterative alignment steps. Evolutionary models were determined, using *ModelFinder* [4] as implemented in *IQTREE* [5], and using *Bayesian Information Criteria* [6] to select the optimal model and gamma rate categories [7]. Subsequently, they used, in successive steps, construction of maximum likelihood phylogenetic trees [8], and further optimization using a *hill-climbing nearest-neighbor interchange* [9] protocol.

To make the cross-species trees, Ho et al*.* [1] used the interactive Fast-Fourier Transform method in MAFFT to build multiple sequence alignments, evolutionary relationships were estimated by *Markov-chain Monte Carlo* [10] in the Bayesian Phylogenetics program and sampling every 1,000 generations in parallel using *the BEAGLE library* [11], following which the within-chain and between-chain variance *potential scale reduction factor* [12] was used to evaluate sufficient sampling. Finally, the sampled posteriors from the two independent executions were combined to generate a *maximum clade-credibility tree* [13]—summarizing the posterior distribution of estimated evolutionary relationships and branch lengths.

This bioinformatics analysis led Ho et al*.* [1] to conclude that type I KRT18 resembles most closely the ancestral precursor of all other type I keratins, and the type II KRT8 resembles most closely the ancestral precursor of all other type II keratins. It is suggested for other gene superfamilies—containing evolutionary blooms in which an ancestral ordering is difficult to resolve—that the comparative genomics approach used in this publication might be helpful in determining which is the earliest diverging gene in a cluster.

Lastly, comparative-genomics approaches on genes relevant to human health and disease can offer insight into the nature and etiology of specific disorders. Are there keratin gene variants known to cause human disease? Ho et al*.* [1] found that the ClinVar database currently lists 26 human disease-causing variants within the various domains of keratin proteins.
